# Dynamic Noise Adaptation in the Motion Model of Monte Carlo Localization for Consistent Localization

**DOI:** 10.3390/s26051415

**Published:** 2026-02-24

**Authors:** Charney Park, Jiyoun Moon

**Affiliations:** Department of Electronics Engineering, Chosun University, Gwangju 61452, Republic of Korea; charney@chosun.kr

**Keywords:** localization, Monte Carlo localization, adaptive motion model, dynamic noise adaptation

## Abstract

Precise position estimation is essential for mobile robots to operate autonomously. In industrial environments that require precision tasks such as docking—including structured indoor facilities such as hospitals, factories, and warehouses—highly accurate localization is often necessary, with accuracy demands ranging from the centimeter to millimeter level depending on the application. Various registration-based localization algorithms have been investigated in response to this requirement. However, fundamental limitations exist, such as a high dependency on initial position estimates, increased computational load, and difficulties in ensuring real-time performance in large-scale environments. The proposed method introduces a dynamic noise adaptation (DNA) technique applicable to the Monte Carlo localization (MCL) algorithm, a particle filter-based localization method, to overcome these limitations. The proposed algorithm improves real-time localization accuracy and estimation consistency by dynamically optimizing the motion noise of MCL using the non-penetration rate, which can serve as a reliability metric in light detection and ranging (LiDAR)-based localization. The proposed algorithm was evaluated in comparison with the expansion Monte Carlo localization 2 (EMCL2) algorithm in both simulation and real-world environments. In the simulated environment, the proposed method achieved lower localization error with respect to the ground truth compared to EMCL2 and the improved adaptive Monte Carlo localization (AMCL) method incorporating a virtual motion model. In real-world experiments, localization performance was evaluated through comparison with a reference trajectory, and the proposed algorithm consistently demonstrated reduced localization error.

## 1. Introduction

Accurate position estimation is crucial for mobile robots to operate autonomously in structured or semi-structured environments. Reliable position information enables robots to perform tasks requiring high precision, such as navigation, manipulation, and docking. Depending on the situation, industrial settings like logistics warehouses or manufacturing lines require centimeter-level and even millimeter-level accuracy [1]. While millimeter-level accuracy is required in certain tightly constrained scenarios, many practical mobile robot applications primarily demand robust and consistent localization performance under varying environmental uncertainties. However, in real-world environments, various uncertainties, such as wheel slip, sensor drift, and changes in ground conditions, occur continuously. These errors accumulate over time, significantly degrading the driving stability and path-following performance [2].

Consequently, registration-based position estimation techniques such as iterative closest point (ICP) [3,4,5] and normal distribution transform (NDT) [6,7] have been extensively studied to address such issues. These methods can achieve high precision by directly registering sensor data with maps; however, they have fundamental limitations, including high dependence on initial position estimates, high computational load due to iterative operations, and difficulty in achieving real-time performance in large-scale environments [8]. Therefore, the application of these registration-based techniques in industrial environments requiring prolonged continuous autonomous driving is limited.

Conversely, Monte Carlo localization (MCL) based on particle filters can effectively handle nonlinear and non-Gaussian error characteristics. In addition, it achieves strengths in global localization by enabling the parallel exploration of various initial position hypotheses [9,10,11,12]. However, the motion model used in conventional MCL generally has the limitation of employing fixed noise parameters [13]. Odometry errors occurring in real-world environments fluctuate constantly depending on the driving mode (straight/turn), floor friction conditions, turning direction, speed, and other factors. When the fixed noise model fails to account for such dynamic uncertainty, particles may converge to incorrect local solutions or produce position estimates that lack consistency in the long term [14].

Recovery techniques, such as sensor resetting and expansion resetting (ER), have been investigated to mitigate these issues [15,16,17]. This approach enables rapid recovery in the event of a fatal error; however, the continuity and consistency of the existing estimated trajectories are compromised when the reset occurs. If used as feedback for driving control, it can degrade path stability. This shows that, although conventional reset-based approaches enhance recovery capabilities, they struggle to sufficiently achieve the consistency essential in environments demanding precise driving.

This study addresses these limitations by introducing a dynamic noise-adaptive MCL motion model. The proposed method improves both localization accuracy and consistency without relying on reset-based recovery. The study contributions can be summarized in three points as follows: First, we introduce the non-penetration rate (NPR) as a new localization reliability metric, which evaluates particle validity based on whether light detection and ranging (LiDAR) scans physically penetrate obstacles on the map. Second, we propose a dynamic noise scaling technique that utilizes NPR to amplify and attenuate noise in the motion model in real time. This effectively expands the particle distribution when localization reliability is low, thereby increasing the likelihood of including the actual location. Third, we compensate for the reduced particle density due to increased noise by integrating the adaptive particle resizing technique, which adaptively adjusts the number of particles in proportion to the noise scale. By combining these three elements, this study proposes a novel MCL framework that improves the accuracy of position estimation during driving. The proposed method was validated through a simulator and real-world experiments.

## 2. Related Work

This section reviews prior research, focusing on position estimation techniques for mobile robots and motion noise modeling in MCL. This process analyzes the structural limitations of conventional approaches and demonstrates the necessity and distinctiveness of the proposed method.

### 2.1. Localization

Mobile robot localization is broadly classified into registration- and probabilistic filter-based methods [9,18]. Registration-based localization techniques, such as ICP [19,20] and NDT [21], compute the position of the robot by directly comparing sensor scan data with a map. These methods have been widely used for high-precision studies, as they can achieve millimeter-level precision under certain conditions. Recent research on registration-based localization has proposed concrete approaches to address computational cost and real-time constraints. Vizzo et al. introduced the KISS-ICP framework, which is based on point-to-point sensor matching to reduce computational complexity while maintaining accuracy [5]. In addition, Kim et al. proposed a Multi-Layered 3D NDT scan matching method to enhance localization robustness in complex environments such as logistics warehouses [6]. McDermott et al. further investigated Online Accuracy Characterization (ICET), which enables scan matching through geometric feature-based analysis [7]. However, they have structural limitations, including a high computational load owing to iterative optimization procedures, a strong dependence on initial position estimation, a lack of real-time capability in large-scale environments, and vulnerability to dynamic obstacles and sensor noise [20]. Consequently, they are not suitable for performing long-term continuous autonomous driving in large and complex areas such as actual industrial environments.

By contrast, particle filter-based MCL [9,10] can efficiently handle nonlinear motion models and non-Gaussian errors and explore various initial position hypotheses in parallel, affording it a strong advantage in global localization [9,10,11,12,22]. MCL estimates the position of a robot by predicting particle states based on odometry and then calculating the likelihood of each particle by comparing its predicted sensor observations with the actual sensor data and known map. This probabilistic structure can resolve the real-time issues of registration-based methods. However, if it fails to accurately reflect the characteristics of odometry errors during the motion update phase, the overall system performance may degrade [10,23].

Despite their advantages, registration-based methods have limitations in real-time performance and continuity. Meanwhile, MCL is limited owing to its incorporation of odometry errors [22,24]. This background suggests the need for dynamic motion noise adjustment techniques capable of reflecting the uncertainty of real-world environments. Therefore, this study proposes a dynamic noise-adaptive MCL to meet these requirements.

### 2.2. Motion Noise in MCL

MCL performance relies significantly on the motion noise model applied during the motion update phase. Thrun et al. modeled odometry errors due to straight motion and rotation as a normal distribution based on four parameters [25,26]. This approach is computationally efficient and widely used in several practical systems, including ROS-based adaptive Monte Carlo localization (AMCL) [13]. However, the odometry errors in real-world environments continuously vary owing to various factors, such as changes in the driving speed, acceleration and deceleration, asymmetry in left and right turns, changes in ground friction conditions, and wheel wear [27]. Fixed noise parameters cannot adequately reflect these non-stationary error characteristics, causing particles to inadequately explore around the actual position and converge to incorrect poses, thereby degrading localization consistency [28]. This loss of consistency can lead to a fatal error in which the particle set completely deviates from its actual position if estimation errors accumulate. Hence, a separate recovery technique is required.

The ER, proposed to address catastrophic localization failure, which involves randomly relocating particles within a specific area to recover from the issue [16,29], is effective for temporary recovery; however, since it is not based on a sensor model, the continuity of the estimated trajectory is disrupted. ER can even deteriorate driving stability if it occurs frequently during normal driving conditions. Several researchers have proposed various reset-based approaches that enhance the handling of fatal errors to address these limitations [15,17]. However, reset-based techniques cannot address the fundamental inaccuracy of motion noise; hence, it still has limitations in terms of improving localization consistency [30,31]. This limitation reveals a fundamental problem: conventional MCL systems cannot account for odometry errors due to changes in dynamic environments. Therefore, we propose an integrated approach that adjusts motion noise in real time, evaluates localization reliability based on particle physical plausibility, and compensates for reduced particle density when noise increases.

### 2.3. Localization Reliability Assessment and Recovery

To address the uncertainty of the motion model in MCL, adaptive techniques based on likelihood statistics and entropy have been widely employed. A representative example is Kullback–Leibler Divergence (KLD) sampling, which dynamically adjusts the number of particles by bounding the statistical error of the particle distribution [32]. However, KLD-based methods primarily contribute to improving computational efficiency through adaptive particle count control and do not actively compensate for the uncertainty inherent in the motion model itself. Therefore, evaluating the reliability of the currently estimated pose is essential. The most reliable indicator for assessing pose estimation accuracy would be sensor-based ground truth. However, because sensor-based ground truth is often difficult to obtain in many environments, a separate metric is required to quantitatively evaluate the reliability of the estimated pose [33]. In previous studies, marginal likelihood has been widely utilized for this purpose [25]. Marginal likelihood is a method that simulates the expected sensor observations at each particle position, calculates the likelihood by comparing it with actual sensor data, and then computes the overall pose reliability based on the weighted sum of all particles. This approach has the advantage of enabling the evaluation of the estimated quality based on sensor models; however, its drawbacks include the computational burden associated with increasing the number of observations and its sensitivity to sensor noise. Furthermore, when the likelihood is excessively concentrated in a specific region, it fails to adequately reflect discrepancies with the actual position, rendering it unsuitable for use as a feedback signal for real-time motion noise control or particle distribution management [32]. Therefore, a new reliability assessment method that reflects the physical validity of position estimation while maintaining computational efficiency is required. For this purpose, this study employs the NPR to evaluate reliability based on the physical validity of LiDAR scans.

The NPR utilizes an algorithm implemented in EMCL2. EMCL2 is based on Ueda’s Expansion Resetting (ER) framework. In the original ER formulation, Ueda introduced the marginal likelihood as a reset trigger, and EMCL1 accordingly employed the marginal likelihood for expansion resetting. However, in contemporary mobile robot localization, LiDAR-based algorithms have become predominant, and the marginal likelihood used in LiDAR-based EMCL1 does not necessarily serve as an optimal reliability indicator. To address this limitation, EMCL2 employs the laser beam-based NPR as the reset trigger. The NPR, which evaluates whether a laser beam penetrates an obstacle, has been applied in previous studies to construct probabilistic occupancy grids in SLAM [34]. NPR exhibits a clear structural difference from marginal likelihood. Unlike approaches that rely on the variance or entropy of a probability distribution, NPR verifies whether LiDAR scan measurements physically penetrate mapped obstacles and uses this physical consistency check as a direct feedback signal. This paper proposes an integrated framework that combines this with motion noise scale adjustment and an adaptive particle count algorithm. Unlike conventional KLD-based methods, which are primarily used to manage particle density based on statistical approximation bounds, the proposed NPR-based scaling actively adjusts the motion model noise parameters in real time when a decrease in localization reliability is detected. Consequently, it evaluates reliability by considering the physical consistency between sensor measurements and the environment and incorporates this information directly into the motion update process. This characteristic distinguishes the proposed approach in that it reflects environmental physical conditions in a stable and responsive manner.

## 3. Method

This study proposes a technique for dynamically adjusting the magnitude of noise distribution within the MCL motion model. We do this by first defining metrics for evaluating localization reliability and presenting a procedure for adjusting the size of the noise distribution based on these metrics. In addition, this section outlines a method for scaling the number of particles to mitigate increased noise distribution.

### 3.1. NPR

In MCL, the sensor model expresses the likelihood of each particle being the actual robot position as a weight. During the sampling step, the average position of particles with higher weights is estimated as the position of the robot. However, evaluating the reliability of these estimated locations requires criteria distinct from weight-based assessments. This study utilizes the NPR as a reliability metric for this purpose. This metric determines whether the measurements acquired by the LiDAR sensor are physically observable at the position of each particle, i.e., they do not penetrate obstacles on the map. If a sensor beam is detected beyond an obstacle on the map, this is considered a physically impossible situation, and the position of the particle is deemed incorrect. Accordingly, the NPR is defined as the proportion of particles that generate physically valid measurements without penetrating obstacles.

(1) $$\phi_{i}=\frac{1}{K}\sum_{k=1}^{K}1\;\left(\mathrm{non}\text{-}\mathrm{penetration}(x_{t}^{\left[i\right]},r_{k},m)\right)$$

(2) $$s_{t}=\frac{1}{N}\sum_{i=1}^{N}\phi_{i}\in \left[0,1\right]$$

Given the scan data set $R_{t}={\left\{r_{k}\right\}}_{k=1}^{K}$, the particle set ${\left\{x_{t}^{\left[i\right]}\right\}}_{i=1}^{N}$, and the map data m, ray casting is performed for each pair (i,k) to evaluate whether an obstacle has been penetrated. If the scan beam *k* emitted from particle *i* does not penetrate an occupied cell of the map grid and terminates near the predicted reach distance ${\hat{r}}_{ik}$, it is judged as a physically valid observation. The penetration status of particle *i* is defined by (Equation (1)), and the NPR at a specific time *t* is expressed by (Equation (2)). The NPR is 1 when no particle penetrates the obstacle and 0 when all particles penetrate it. A value closer to 1 indicates higher reliability in the estimated localized position at that time, whereas a value closer to 0 indicates lower reliability. Because the NPR metric evaluates sensor-map consistency based on physical penetration of mapped occupied regions, transient dynamic obstacles such as pedestrians or other robots typically have limited influence unless they induce systematic violations of the assumed map geometry.

### 3.2. Motion Model Noise Parameter Scaling

This study proposes a motion model noise parameter scaling technique to dynamically adjust the magnitude of noise distribution by utilizing the NPR as a feedback signal. In the MCL motion model, the position of the particle is updated during robot movement through dead-reckoning-based motion estimation. However, moving all particles with the same dead-reckoning value makes it difficult to account for errors arising from non-ideal factors such as wheel slip. Therefore, additional noise is typically added to compensate for this. This study employs a normal distribution-based probabilistic error for this noise. When the odometry increments are denoted as δrot1, δtrans, and δrot2, these noises are expressed as $\sigma_{rot1}^{2}$, $\sigma_{trans}^{2}$, and $\sigma_{rot2}^{2}$, respectively, as shown in Equation (3).

(3) $$\begin{array}{c} \;\delta_{rot1}∼N({\hat{\delta }}_{rot1},\sigma_{rot1}^{2}),\;\sigma_{rot1}^{2}=\alpha_{1}{\hat{\delta }}_{rot1}^{2}+\alpha_{2}{\hat{\delta }}_{trans}^{2},\\ \delta_{trans}∼N({\hat{\delta }}_{trans},\sigma_{trans}^{2}),\;\sigma_{trans}^{2}=\alpha_{3}{\hat{\delta }}_{trans}^{2}+\alpha_{4}\left({\hat{\delta }}_{rot1}^{2}+{\hat{\delta }}_{rot2}^{2}\right),\\ \;\delta_{rot2}∼N({\hat{\delta }}_{rot2},\sigma_{rot2}^{2}),\;\sigma_{rot2}^{2}=\alpha_{1}{\hat{\delta }}_{rot2}^{2}+\alpha_{2}{\hat{\delta }}_{trans}^{2}. \end{array}$$

Since noise characteristics differ depending on the driving direction of the robot, forward and rotational errors occurring during forward and rotational driving, respectively, are separated and defined as four distinct noise components. In a typical MCL algorithm, these four noise metrics are parameterized and utilized to appropriately adjust these noise characteristics for the robot platform. This study adopts a method of applying scaling to each noise parameter to adjust the noise level in real time. When localization reliability is at its maximum, the existing parameter values are used as-is. As reliability decreases, scaling is applied to the parameter values. The scaling factor used at this point is defined by Equation (4). The parameter β controls the gain of the linear scaling factor and determines how sensitively the motion noise parameters respond to changes in localization reliability. Larger values of β lead to a more aggressive increase in motion noise as the NPR decreases, which can improve robustness under severe odometry errors but may also reduce localization stability due to excessive particle dispersion. Conversely, smaller values result in more conservative noise scaling, preserving stability at the cost of reduced adaptability. In this study, β was fixed to 1.0 in all experiments, providing a balanced trade-off between responsiveness and stability without causing abrupt noise inflation.

(4) $$\lambda_{\mathrm{lin}}\left(s_{t}\right)=1+\beta \left(1-s_{t}\right),\;\beta >0$$

(5) $${\tilde{\alpha }}_{j}\left(t\right)=\lambda_{\mathrm{lin}}\left(s_{t}\right)\alpha_{j}\;\left(j=1,2,3,4\right)$$

(6) $$\begin{array}{cc} {\tilde{\sigma }}_{\mathrm{rot}1\;}^{2} & ={\tilde{\alpha }}_{1}{\hat{\delta }}_{\mathrm{rot}1\;}^{2}+{\tilde{\alpha }}_{2}{\hat{\delta }}_{\mathrm{trans}\;}^{2},\\ {\tilde{\sigma }}_{\mathrm{trans}\;}^{2} & ={\tilde{\alpha }}_{3}{\hat{\delta }}_{\mathrm{trans}\;}^{2}+{\tilde{\alpha }}_{4}\left({\hat{\delta }}_{\mathrm{rot}1\;}^{2}+{\hat{\delta }}_{\mathrm{rot}2\;}^{2}\right),\\ {\tilde{\sigma }}_{\mathrm{rot}2\;}^{2} & ={\tilde{\alpha }}_{1}{\hat{\delta }}_{\mathrm{rot}2\;}^{2}+{\tilde{\alpha }}_{2}{\hat{\delta }}_{\mathrm{trans}\;}^{2}. \end{array}$$

In this framework, the motion noise parameters $\alpha_{1}$–$\alpha_{4}$ are fixed base parameters that characterize the nominal odometry uncertainty of the robot platform. At each time step, the current NPR value is computed as described in Section 3.1 and used to derive a scaling factor. Using this factor, the scaled noise parameters are recomputed at every motion update step and incorporated into the motion model as shown in Equations (5) and (6). The scaled parameters are applied only during the sampling process of the motion model, while the base parameters remain unchanged. This procedure increases the noise distribution in real time when localization reliability decreases, thereby enhancing the probability that the particle set includes the actual robot position.

While the same scaling factor is uniformly applied to all motion noise parameters, the proposed method does not aim to distinguish the error source through the scaling process itself. Instead, the distinction between different motion error sources is already encoded in the underlying parameter $\alpha_{1}$–$\alpha_{4}$, indicating the relative contribution of translational and rotational uncertainties to the kinematic properties of the robot. The NPR-based scaling factor reflects global position reliability measurements rather than directional or component-specific error information. Thus, scaling all motion noise parameters uniformly allows us to adjust the magnitude of the overall uncertainty while maintaining a predefined relative structure between various error sources. This design choice ensures stable and consistent behavior of the motion model without additional tuning variables under complex motion patterns. While parameter-specific scaling can further differentiate error sources, it requires additional reliability indicators beyond NPR and can increase the risk of tuning complexity and unstable particle dispersion. For this reason, this study adopts a single reliability-based scaling factor to balance robustness, stability, and general applicability.

Several prior studies have proposed adaptive mechanisms in Monte Carlo Localization, primarily focusing on particle number adjustment, resampling strategies, or sensor model adaptation based on likelihood statistics or entropy measures. In these approaches, the motion noise parameters are typically treated as fixed or tuned offline. In contrast, the proposed method introduces an online adaptation mechanism directly within the motion model by scaling motion noise parameters according to the NPR metric, which captures sensor-map consistency. This allows the motion uncertainty to be adjusted dynamically in response to localization reliability while preserving the simplicity and general applicability of the standard MCL framework.

### 3.3. Adaptive Resize of Particles

As noise distribution increases, the spatial range in which particles can be distributed expands; however, this leads to a corresponding decrease in particle density. This study addresses this issue by employing a method that increases the number of particles as the noise distribution expands. The target particle number is defined as in Equation (7). The parameter γ controls the sensitivity of the particle resizing mechanism to changes in localization reliability. Specifically, γ determines how aggressively the number of particles is adjusted in response to variations in the NPR, thereby regulating the balance between computational efficiency and localization robustness. Larger values of γ result in more frequent and pronounced particle resizing under degraded reliability, which can enhance recovery from localization degradation but may increase computational load and particle dispersion. Conversely, smaller values of γ lead to more conservative resizing behavior, maintaining computational stability at the expense of reduced adaptability to severe uncertainty.

In this study, γ was fixed to a constant value across all experiments to ensure consistent behavior and to avoid environment-specific tuning. The selected value provided stable particle set adaptation without inducing excessive fluctuations in particle count.

(7) $$N_{t}=\mathrm{clip}\left(\left\lceil N_{\min}\lambda {\left(s_{t}\right)}^{\gamma }\right\rceil ,N_{\min},N_{\max}\right),\;\gamma \in \left[0.5,\;1.0\right]$$

The process of increasing the number of particles follows the approach of the conventional AMCL. Once the target particle count is determined, multinomial resampling is performed on the existing particle set to replicate particles up to the target number. This process is performed only when the number of particles fluctuates. Owing to the nature of multinomial resampling, the same particle may be replicated multiple times; however, since noise is subsequently applied in the motion model, this may induce the particle distribution over a wide area, which aligns with the original intent.

### 3.4. Dynamic Noise Adaptation (DNA) Pipeline

This section describes the procedure for applying the previously proposed non-penetration rate-based motion model noise parameter scaling technique and adaptive particle resizing technique to MCL. In addition, it presents the pipeline for the entire localization system.

**Figure 1:**
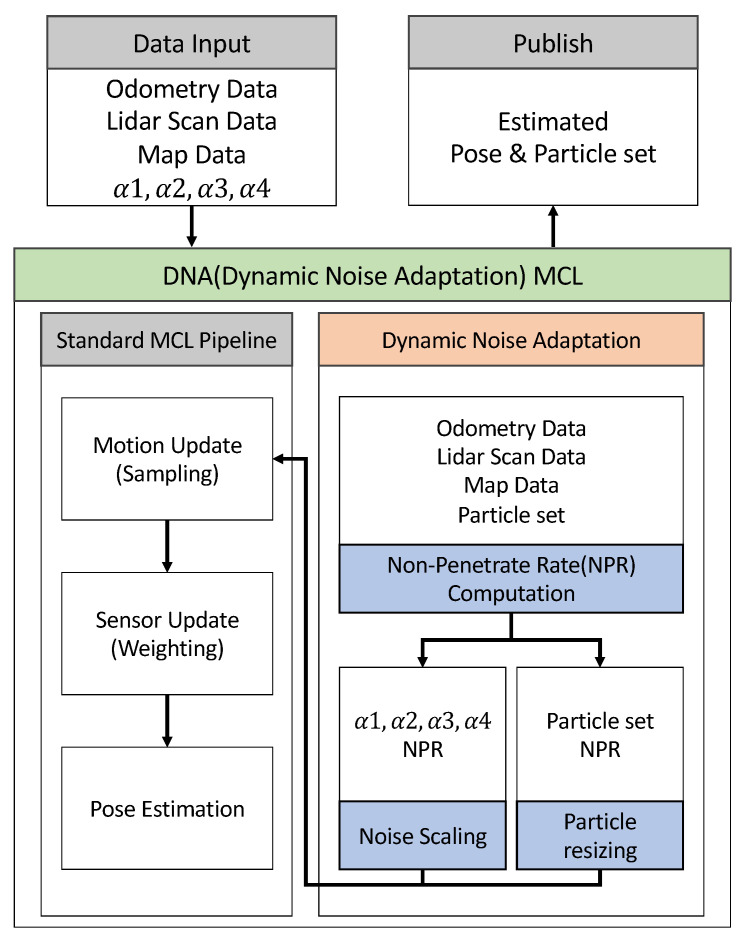
Overall architecture of the proposed dynamic noise-adaptive Monte Carlo localization framework based on NPR and motion model noise scaling.

The overall structure can be seen in Figure 1. The input data for this system comprises odometry, LiDAR scans, and map data. The data are utilized to perform the fundamental steps of MCL: motion update, sensor update, and pose estimation. The noise parameter used in the motion model is dynamically adjusted via the proposed motion model noise parameter scaling, and the additional particles generated during the adaptive resize of particles step are subsequently incorporated into the motion model process. The subsequent sensor update and pose estimation stages follow the standard MCL pipeline. The overall operational procedure of the algorithm is summarized in Algorithm 1.

Unlike reset-based position estimation algorithms such as EMCL2, which use reliability indicators only as reset triggers, the proposed pipeline uses reliability indicators as direct feedback to motion updates. NPR is calculated for each time step and is used as an indicator to adjust both motion noise parameters and particle set size before the sampling step. As a result, errors can be continuously and actively reduced during localization. Through this structure, the proposed method can reduce the odometry error that changes over time without interfering with the continuity of the trajectory.

Within the pipeline, dynamic noise adaptation operates in parallel with the standard MCL process and does not change the order of existing motion updates, sensor updates, and pose estimation stages. The NPR calculation relies only on the information already in use in the localization system, including current particle sets, LiDAR measurements, and static maps. As a result, dynamic noise adaptation is applied only to the motion update stage by adjusting the noise parameters and particle count. This modular structure can be applied while maintaining compatibility with general-purpose MCL.
**Algorithm 1:** Pseudocode of the proposed dynamic noise-adaptive MCL algorithm**Input**:  map *m*, particles ${\left\{x_{t-1}^{\left[i\right]},\;w_{t-1}^{\left[i\right]}\right\}}_{i=1}^{N_{t-1}}$, control $u_{t}$, scan $z_{t}$**Params**:  $\alpha_{1}..\alpha_{4}$, β, γ, $N_{\min}$, $N_{\max}$, $s_{\mathrm{ER}}$, $L_{\mathrm{ER}}$, smoothing η  1: // Data Input & Initialization (Figure 1: Data Input)  2: $X_{t-1}\leftarrow {\left\{x_{t-1}^{\left[i\right]},\;w_{t-1}^{\left[i\right]}\right\}}_{i=1}^{N_{t-1}}$  3:  4: // Dynamic Noise Adaptation branch: NPR & Noise Scaling (Figure 1, right block)  5: $s_{t}\leftarrow \mathrm{NPR}\left(\left\{x_{t-1}^{\left[i\right]}\right\},\;z_{t},\;m\right)$    // via raycasting & Equations (1) and (2)  6: ${\bar{s}}_{t}\leftarrow \left(1-\eta \right)\;{\bar{s}}_{t-1}+\eta \;s_{t}$    // temporal smoothing of NPR  7: $\lambda_{t}\leftarrow \lambda \left({\bar{s}}_{t}\right)$ using Equation (4);    ${\tilde{\alpha }}_{j}\leftarrow \lambda_{t}\cdot \alpha_{j}$ for j=1..4    // Equations (5) and (6)  8:  9: // Standard MCL Pipeline: Motion Update (Sampling) (Figure 1, left block)10: **for** $i=1..N_{t-1}$ **do**11:    sample $\left(\delta_{\mathrm{rot}1},\;\delta_{\mathrm{trans}},\;\delta_{\mathrm{rot}2}\right)$12:       with variances computed from ${\tilde{\alpha }}_{j}$    // scaled motion noise, Equation (3)13:    $x_{t}^{\left[i\right]}\leftarrow \mathrm{motion}\text{\_}\mathrm{update}\;\left(x_{t-1}^{\left[i\right]},\;\delta_{\mathrm{rot}1},\;\delta_{\mathrm{trans}},\;\delta_{\mathrm{rot}2}\right)$14: **end for**15:16: // Standard MCL Pipeline: Sensor Update (Weighting)17: **for** $i=1..N_{t-1}$ **do**18:    $w_{t}^{\left[i\right]}\leftarrow p\left(z_{t}|x_{t}^{\left[i\right]},\;m\right)$19: **end for**20: normalize $\left\{w_{t}^{\left[i\right]}\right\}$21:22: // Standard MCL Pipeline: Pose Estimation23: ${\hat{x}}_{t}\leftarrow \mathrm{PoseEstimate}\;\left(\{x_{t}^{\left[i\right]},\;w_{t}^{\left[i\right]}\}\right)$    // e.g., weighted mean or MAP24:25: // Dynamic Noise Adaptation branch: Particle Resizing (Figure 1, “Particle Resizing”)26: $N_{t}\leftarrow \mathrm{clip}\;\left(\left\lceil N_{\min}\cdot \lambda_{t}^{\gamma }\right\rceil ,\;N_{\min},\;N_{\max}\right)$    // Equation (7)27: $X_{t}\leftarrow \mathrm{Resample}\;\left(\left\{x_{t}^{\left[i\right]},\;w_{t}^{\left[i\right]}\right\},\;N_{t}\right)$    // low-variance or multinomial28:29: // Fallback Expansion Resetting (rare; outside main pipeline path)30: **if** $\left({\bar{s}}_{t}<s_{\mathrm{ER}}\right)$ **and** $\left(\sum_{i}w_{t}^{\left[i\right]}<L_{\mathrm{ER}}\right)$ **then**31:    $X_{t}\leftarrow \mathrm{UniformResetBox}\;\left(\mathrm{center}=\arg\max_{i}w_{t}^{\left[i\right]},\;\mathrm{size}=\mathrm{predef}\right)$32: **end if**33:34: // Publish block (Figure 1, “Publish”)35: $\mathrm{Publish}\;\left({\hat{x}}_{t},\;X_{t}\right)$    // publish estimated pose & particle set36: **return** $\left({\hat{x}}_{t},\;X_{t}\right)$

From a computational perspective, the proposed method does not modify the fundamental structure of the Monte Carlo Localization algorithm. The additional operations introduced by the NPR-based adaptation consist of computing the NPR metric and applying a scalar scaling factor to the motion noise parameters, both of which scale linearly with the number of particles. As a result, the overall computational complexity remains equivalent to that of standard MCL, and the additional overhead is negligible compared to sensor likelihood evaluation and resampling steps.

## 4. Experimental

A series of experiments was conducted to evaluate the performance of the proposed algorithm. For comparison, the EMCL2 algorithm and an improved AMCL algorithm incorporating a virtual motion model were employed. Although EMCL2 does not directly contribute to improving real-time localization accuracy, it is known for its effective recovery performance in fatal localization failure scenarios through the use of Expansion Resetting (ER). The improved AMCL algorithm used in the experiments was proposed in 2025 and was developed to address limitations of conventional MCL frameworks in which motion updates are not available under stationary conditions [22]. To enable the motion model even under stationary conditions, the algorithm evaluates scan matching quality based on Normal Distributions Transform (NDT) and applies virtual odometry toward locations with high matching scores. This approach contributes to improved convergence speed during initial cold-start scenarios and enhances localization accuracy when the robot remains stationary.

To enable a controlled evaluation of the proposed DNA mechanism within a consistent Monte Carlo Localization framework, EMCL2 was used as the baseline algorithm. The NPR-based dynamic motion noise, adaptive particle resizing, and virtual motion model components were implemented separately. Each component was selectively activated to allow systematic comparison. The experiments were conducted in both simulated and real-world environments. In the simulated environment, performance was evaluated with respect to the ground truth. In the real-world environment, the estimated localization trajectories were compared with a reference trajectory.

### 4.1. Experimental Robot Platform

**Figure 2:**
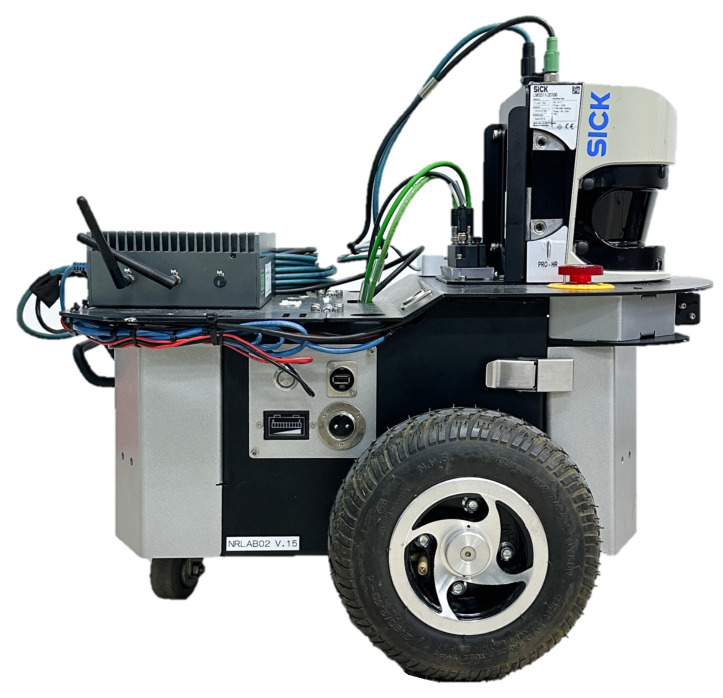
NRLAB02 differential-drive mobile robot platform equipped with a 2D LiDAR, inertial measurement unit, and wheel odometry sensors.

This study utilized RedOne Technology’s NRLAB02 robot (RedOne Technology, Jangseong-gun, Republic of Korea) as the experimental platform. NRLAB02 is based on a two-wheel differential-drive structure and is equipped with sensors necessary for localization, including 2D LIDAR, an inertial measurement unit, and wheel encoders. A photograph of the NRLAB02 mobile robot platform is provided in Figure 2. The same robot model was applied in both the simulated and real environments by modeling the robot using the unified robot description format (URDF). The URDF file contains physical parameters, including the wheel radius and wheelbase, wheel drive range, mesh collider for the robot surface, and mass information for each component. Furthermore, the robot control system was configured based on Robot Operating System 2 (ROS2), and both the proposed and comparison algorithms were run in the same system environment.

### 4.2. Simulator Environment

**Figure 3:**
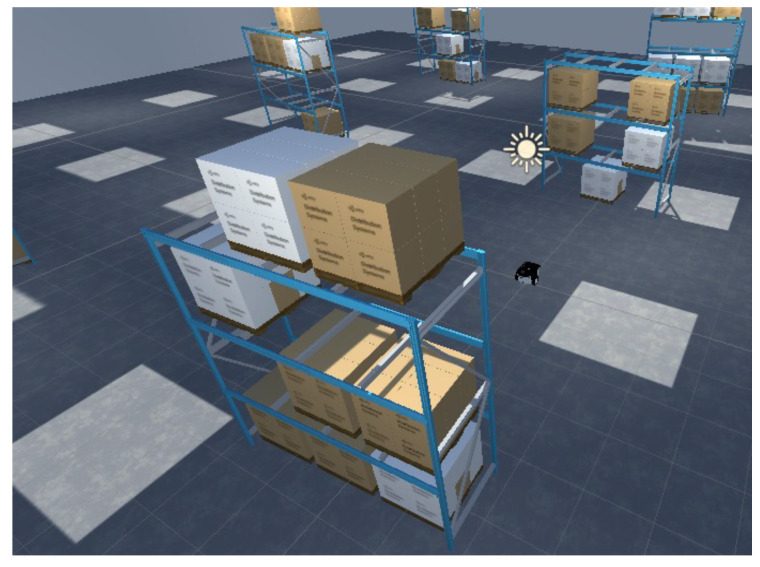
Simulator environment representing a factory-like warehouse scenario.

The simulator environment was built using the Unity 3D engine. The driving environment utilized the warehouse provided by Unity, which is a simplified representation of an actual logistics center. The environment features shelves and boxes randomly placed inside a square-shaped building. Physical interactions between robots and the environment were handled via Unity’s physics engine, and robot models based on mesh colliders were created within the Unity environment using the URDF. By assigning physical characteristics identical to those of an actual robot—such as wheel-driven friction, the mass of each part, and the wheel’s range of motion—we constructed a physics-based simulator environment capable of conducting experiments on mobile driving robots. A rendered view of the simulator environment is shown in Figure 3.

**Figure 4:**
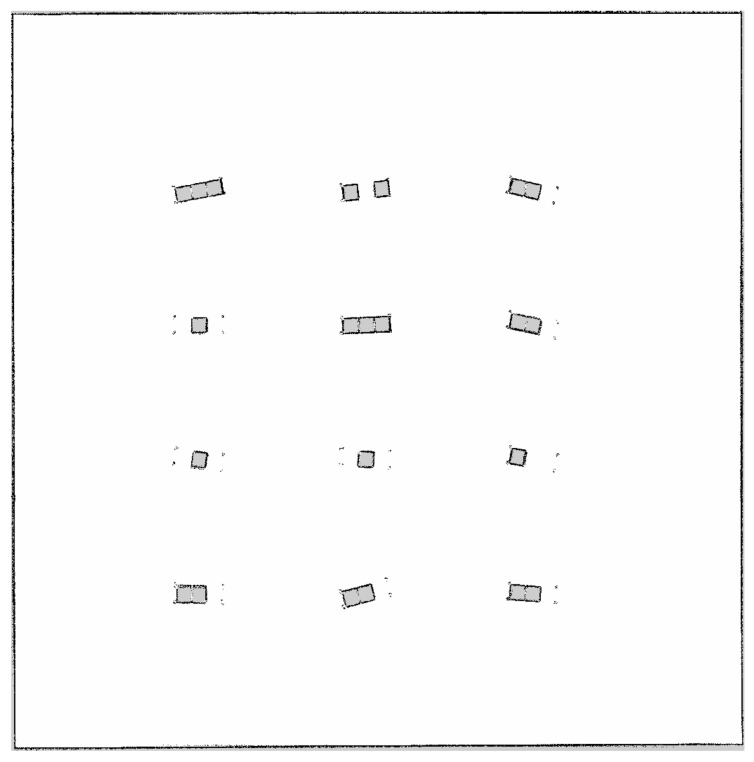
Occupancy grid map of the simulator environment with a resolution of 0.05 m and irregularly aligned shelves.

The robot control and sensor data reception interface was implemented based on ROS2. Each sensor was configured to faithfully replicate the operating principles of the sensors mounted on the actual robot. The LIDAR sensor was implemented by emitting beams of precise specifications via ray casting from positions defined in the URDF, measuring the distance to the first collider object encountered. In addition, we added a feature to output ground truth information for the performance verification of the proposed algorithm. The ground truth was calculated by converting the absolute position of the robot object provided by the Unity engine into the map coordinate system. In this study, we generated an environmental map using the Slam Toolbox. The map resolution was set to 0.05 m, enabling the construction of a precise map covering the entire driving path. The occupancy grid map of the simulator environment is shown in Figure 4.

### 4.3. Real-World Environment

**Figure 5:**
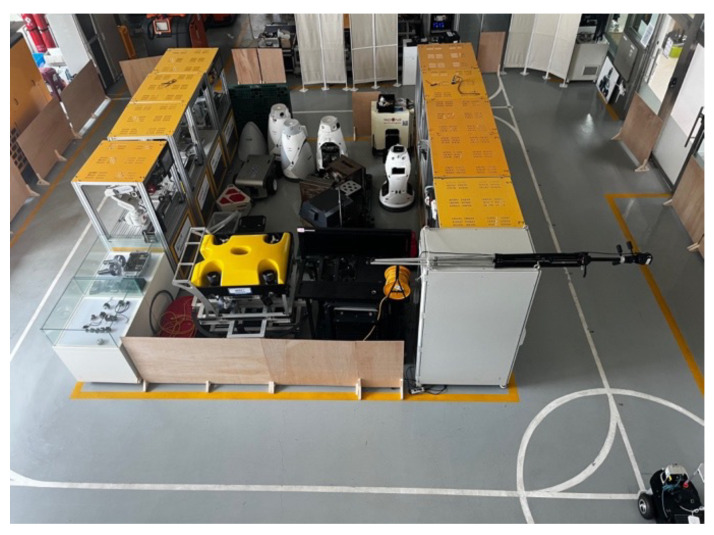
Real-world factory-like environment used for the localization experiments.

**Figure 6:**
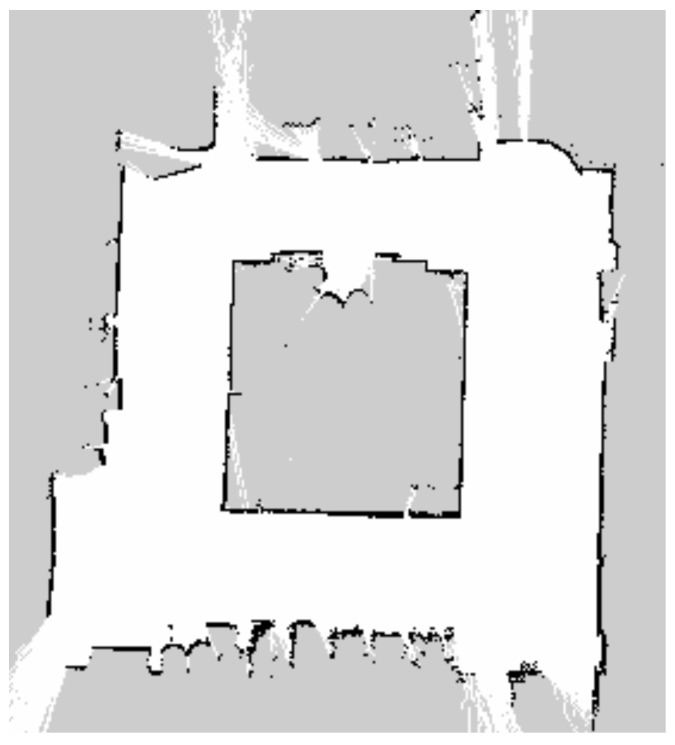
Occupancy grid map of the real-world factory-like experimental environment with a resolution of 0.05 m.

The real-world experiments were conducted in two environments. The first environment was designed to simulate an indoor factory environment. The floor comprised epoxy-coated concrete, which causes wheel slip and introduces noise into the odometry data. In addition, it includes walls and obstacles made of various materials, such as metal shelves, plastic boxes, and glass partitions. In particular, glass and stainless steel frames generate strong noise in LIDAR scan data, creating an environment where localization is difficult. The test section was designed to include narrow passages and 90° intersections, thereby enabling a comparison of the localization performance during straight driving and turning maneuvers. A photograph of the factory-like real-world environment is shown in Figure 5. The map used in the experiment was generated using the Slam Toolbox, identical to the simulator environment, with a resolution set to 0.05 m. Furthermore, similar to the simulator environment, the entire path was traversed at least twice during map generation to ensure the loop closure function of Slam Toolbox, a graph-based SLAM method, performed correctly. The corresponding occupancy grid map of this environment is shown in Figure 6.

**Figure 7:**
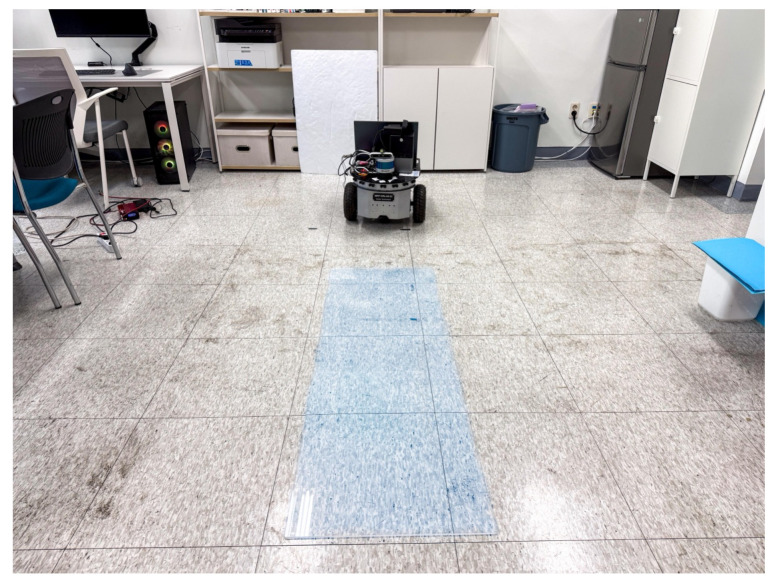
Narrow indoor experimental environment incorporating an extremely low-friction surface.

**Figure 8:**
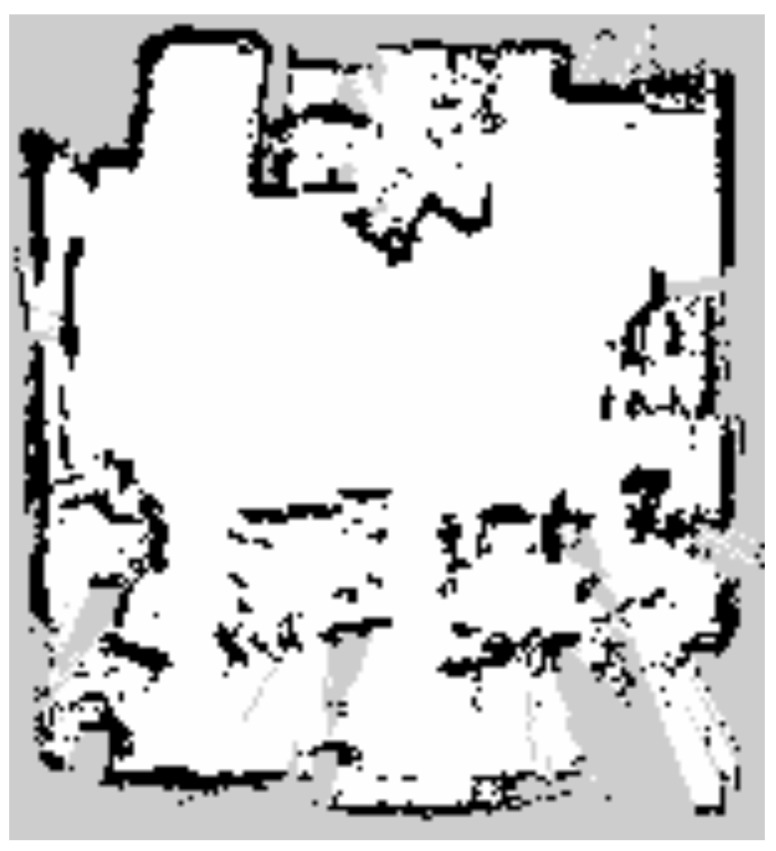
Occupancy grid map used in the narrow indoor environment with a resolution of 0.05 m.

The second environment was constructed to simulate a narrow indoor space in which household and indoor service robots typically operate. To reproduce uncontrolled surface conditions characterized by continuously varying wheel slip, the environment was composed of multiple surface materials. Approximately half of the driving area was covered with smooth acrylic panels, and soapy water was applied to create a wet, low-friction surface. Owing to the non-uniform friction characteristics of the driving surface, the reliability of raw odometry data collected from wheel encoders is reduced. These abnormal surface conditions increase motion model uncertainty and provide an experimental environment suitable for evaluating localization robustness. A photograph of the narrow indoor experimental environment is shown in Figure 7. The map used in this experiment was generated using SLAM Toolbox, consistent with the previous environments, and the map resolution was set to 0.05 m. Following the map generation process commonly used for consumer-grade robots, such as robot vacuum cleaners and companion robots, the map was created by traversing the entire area only once. The occupancy grid map of the narrow indoor environment is shown in Figure 8. By employing diverse surface conditions and a map with relatively low precision, this environment enables evaluation of the proposed method under conditions representative of real-world consumer product applications.

Because accurate ground truth data were difficult to obtain in both real-world environments, a quantitative comparison based on ground truth was not conducted. Instead, the experimental evaluation was performed by comparing a reference driving trajectory with the localization results obtained using the proposed dynamic noise adaptation technique, the baseline EMCL2 algorithm without this mechanism, and EMCL2 incorporating a virtual motion model.

### 4.4. Experimental Procedure

In this experiment, the robot was driven along a predefined path while recording sensor data. The same recorded data was then input into each algorithm to compare the localization results. This enables a fair comparison of algorithm performance under completely identical sensor input conditions and allows the evaluation of improvements in localization accuracy and stability across different scenarios. The recorded sensor data included LIDAR and encoder data. In the simulated environment, ground truth data were recorded during robot motion, enabling a more accurate comparison of localization performance. In contrast, ground-truth-level data were not recorded in the real-world environment. Instead, the robot was driven using a precision control controller with ICP-based feedback to follow a reference trajectory, and the experimental results were evaluated with respect to this reference trajectory.

**Figure 9:**
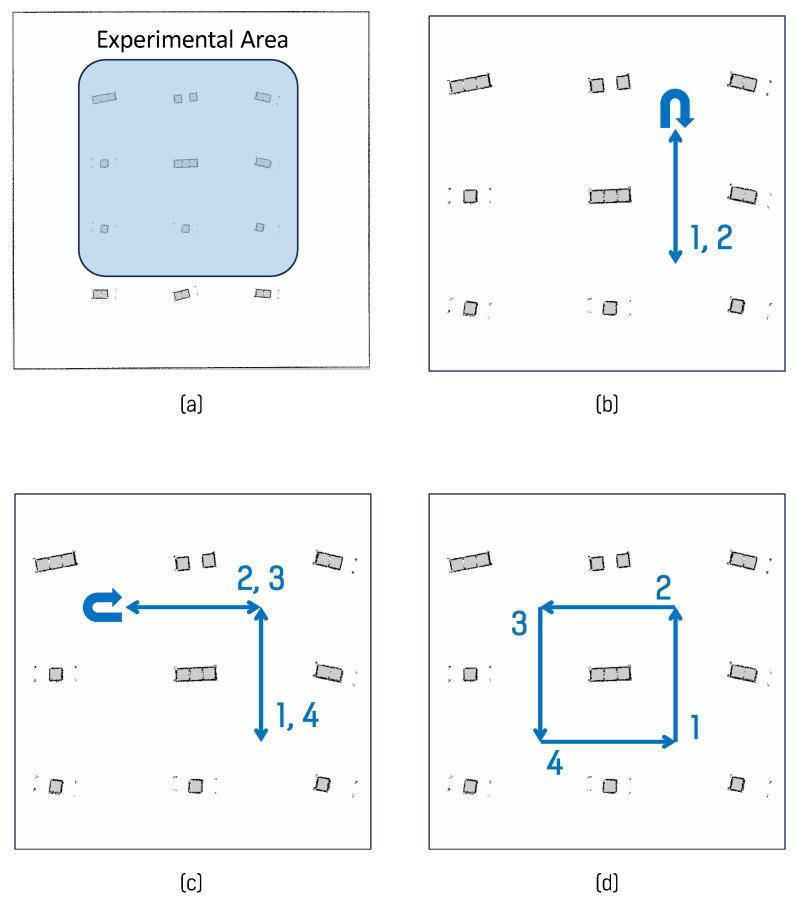
Experimental paths in the simulator environment: (**a**) experimental area, (**b**) I-shaped path, (**c**) L-shaped path, (**d**) square path. The arrows indicate the driving direction, and the numbers denote the order of repeated traversal along each path.

**Figure 10:**
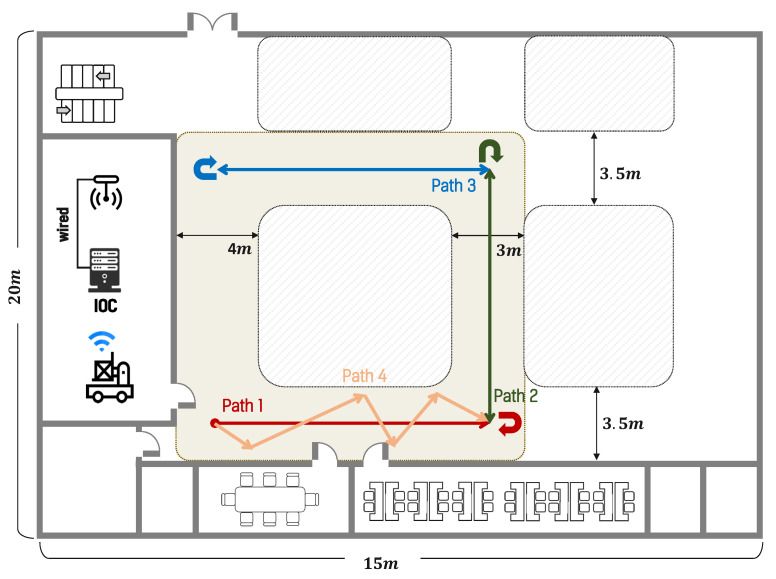
Experimental paths in the factory-like environment: Path 1 (I-shaped), Path 2 (L-shaped), Path 3 (U-shaped with right-angle turns), and Path 4 (unstructured path). Arrows indicate the driving direction of the robot along each path.

**Figure 11:**
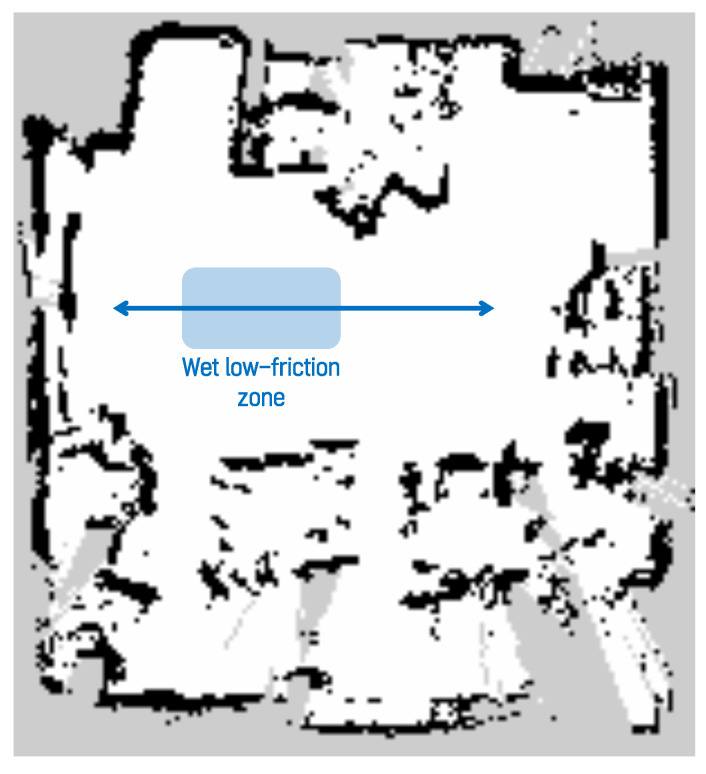
Experimental driving path in the narrow indoor environment. The arrow indicates the driving direction of the robot.

We utilized three paths in the simulated environment, as shown in Figure 9, in the factory-like environment, four distinct paths were defined, as shown in Figure 10. Finally, in the narrow indoor simulated environment, a path was designed in which the robot moved straight ahead, performed a turn, and returned to the starting point, as shown in Figure 11. In the simulator environment, each path was driven five times under identical conditions. In the narrow indoor simulated environment, the same path was traversed ten times to ensure that the effects of wheel slip on the low-friction surface were sufficiently reflected. During path following, the robot’s heading was adjusted to point toward intermediate waypoints. In sections where paths overlapped, it was programmed to perform a 180° turn, enabling performance evaluation across different driving scenarios.

In addition, experiments were conducted to analyze the effects of different β and γ values. These experiments were performed in the simulator environment, where RMSE with respect to the ground truth was measured. For β, the values 0.5, 1.0, and 1.5 were compared, while for γ, the values 0.5 and 1.0 were evaluated.

### 4.5. Experimental Results

The experiments were broadly conducted in two settings: a simulated environment and real-world environments. In the simulation environment, ground truth data were utilized to compare the proposed algorithm with EMCL2 and a virtual motion model-based algorithm, and performance was quantitatively evaluated using MAE and RMSE. In the real-world experiments, tests were performed in a factory-like environment and a narrow indoor low-friction environment. Because ground truth was difficult to obtain, localization performance was analyzed by comparing the estimated trajectories with a reference trajectory.

#### 4.5.1. Simulation Results

**Figure 12:**
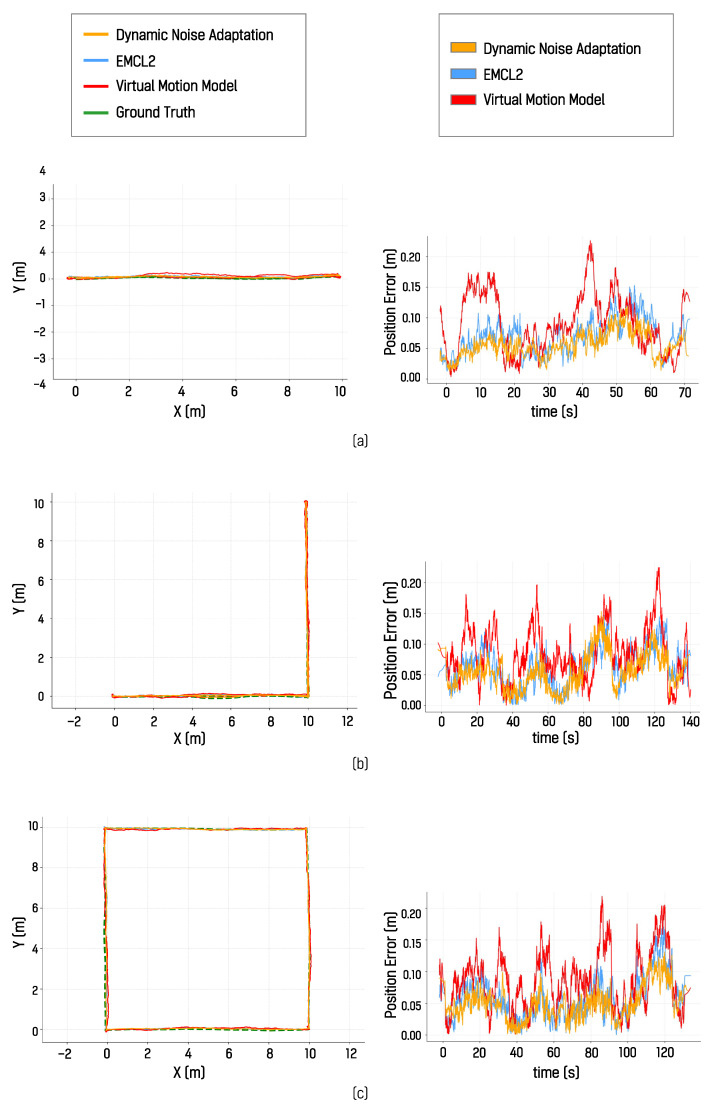
Localization results in the simulator environment showing estimated trajectories and position errors over time: (**a**) I-shaped, (**b**) L-shaped, and (**c**) square paths.

These experimental results show that, in the simulated environment, the algorithm incorporating dynamic noise adaptation showed lower localization error than both the EMCL2 algorithm and the virtual motion model-based algorithm. Across all experiments in which three different paths were each traversed five times, the algorithm with dynamic noise adaptation consistently produced the smallest error with respect to the ground truth. Quantitative localization errors for all trajectories and runs are summarized in Table 1. For each path, the estimated localization pose trajectory from the first trial and the corresponding position error over time relative to the ground truth are shown in Figure 12.

**Figure 13:**
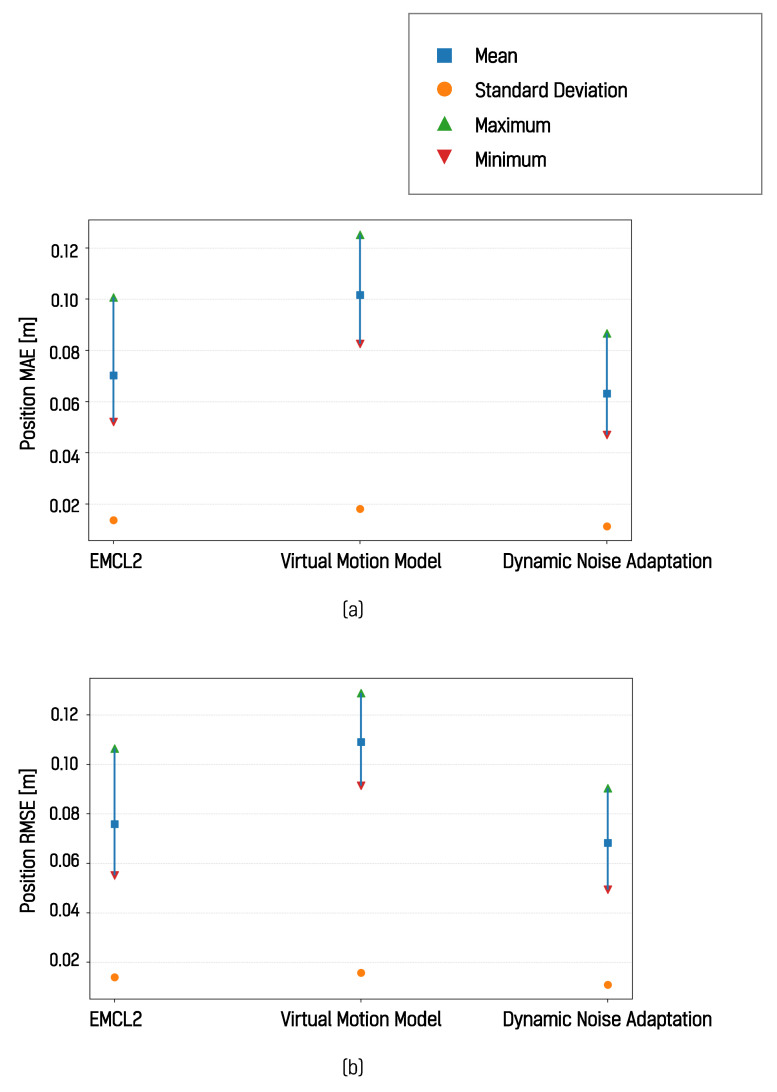
Comparison of position MAE and position RMSE: (**a**) MAE, (**b**) RMSE.

The results for the I-shaped path in Figure 12a indicate lower error and higher estimation consistency. The results for the L-shaped path in Figure 12b show similar temporal trends in error variation, while the results for the square path in Figure 12c demonstrate that the localization error was reduced in all four segments when dynamic motion noise adaptation was applied. These results were further summarized and compared using mean absolute error (MAE) and root mean square error (RMSE), as shown in Figure 13.

The application of the DNA algorithm resulted in an average RMSE reduction of 0.0094 m. Both the mean and maximum RMSE values were observed to be lower when the proposed method was applied. Under these experimental conditions, the virtual motion model showed the largest localization error among the evaluated methods. This observation shows that, in simulated environments with consistent surface conditions and reliable wheel odometry, virtual odometry does not necessarily contribute to improved localization accuracy.

#### 4.5.2. Real-World Results I: Factory-like

**Figure 14:**
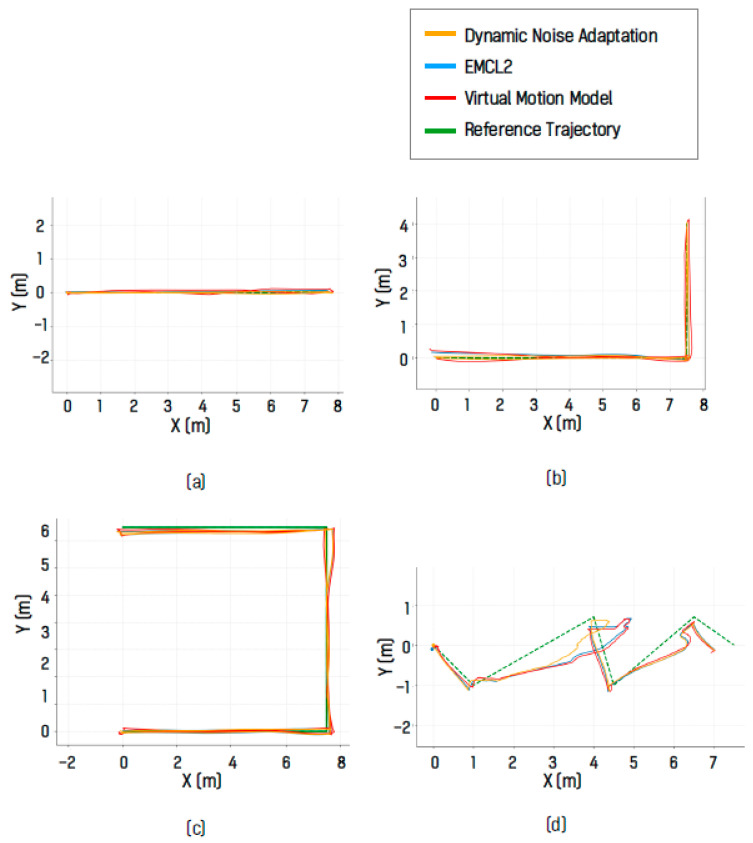
Localization results in the real-world environment showing estimated trajectories: (**a**) I-shaped, (**b**) L-shaped, and (**c**) U-shaped paths, (**d**) unstructured paths.

In the factory-like environment, the localization pose trajectories obtained using the algorithm with dynamic motion noise adaptation, the EMCL2 algorithm, and the virtual motion model-based algorithm were compared with a reference trajectory. The estimated localization pose trajectories for each driving trial are shown in Figure 14. In Figure 14a–c, the DNA algorithm produced trajectories that most closely followed the reference trajectory. For the unstructured path shown in Figure 14d, only the localization pose trajectory generated by the DNA algorithm approached the reference trajectory at the rotation waypoint.

**Figure 15:**
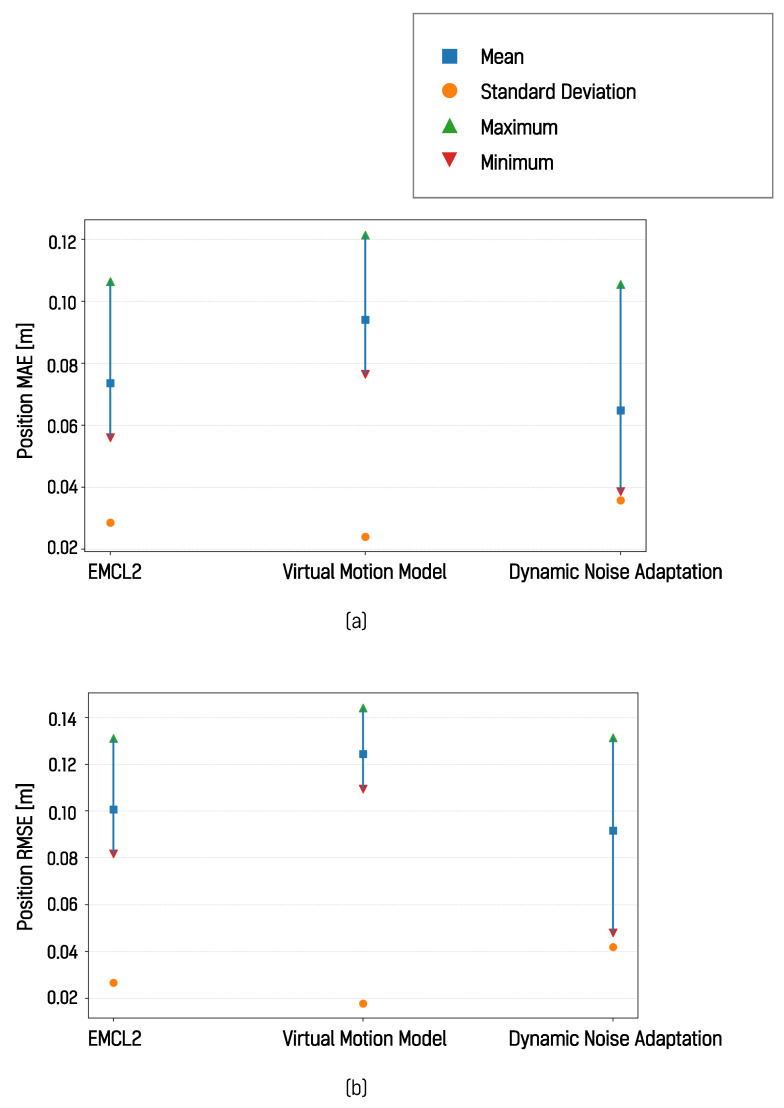
MAE and RMSE calculated with respect to the reference trajectory in the factory-like environment: (**a**) MAE, (**b**) RMSE.

In addition, the maximum deviation from the reference trajectory was observed to be smaller for the DNA algorithm. To quantify the localization error relative to the reference trajectory, the MAE and RMSE were computed for all trials and are presented in Table 2 and Figure 15. The average MAE values were 0.1288 m for EMCL2, 0.1450 m for the virtual motion model, and 0.1226 m for DNA. The average RMSE values were 0.1766 m for EMCL2, 0.1931 m for the virtual motion model, and 0.1683 m for DNA. These results show that the DNA algorithm achieved the lowest average MAE and RMSE values among the compared methods.

#### 4.5.3. Real-World Results II: Narrow Indoor/Low-Friction

**Figure 16:**
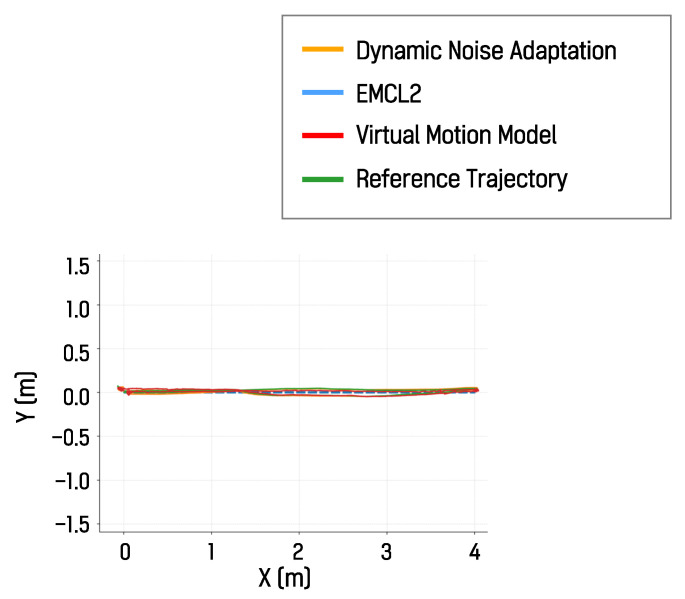
Localization trajectories in the narrow indoor real-world environment.

**Figure 17:**
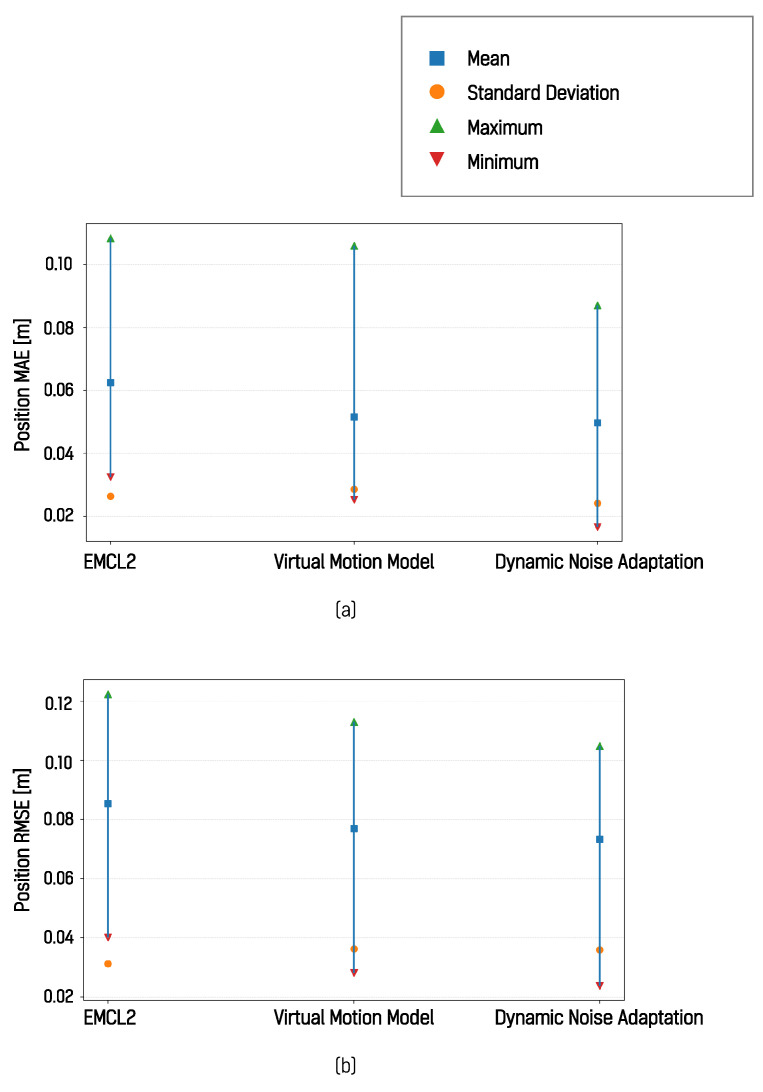
Localization error of each algorithm with respect to the reference trajectory in the narrow indoor real-world environment: (**a**) MAE, (**b**) RMSE.

In the narrow indoor real-world environment, the localization pose trajectories produced by the algorithm with dynamic motion noise adaptation, the EMCL2 algorithm, and the virtual motion model-based algorithm were compared with a reference trajectory. One representative result from the ten driving trials is shown in Figure 16. The MAE and RMSE values computed with respect to the reference trajectory are presented in Table 3 and Figure 17. In this environment, the DNA algorithm achieved the lowest average MAE and RMSE values, followed by the virtual motion model-based algorithm. These results suggest that, when the driving environment includes surfaces that induce substantial wheel slip, techniques such as dynamic noise adaptation and virtual motion models can be effective in improving localization performance. These experimental results show that the proposed real-time dynamic noise adaptation technique can reduce localization error across various environments compared with the reset-based EMCL2 approach and the virtual motion model-based technique.

#### 4.5.4. Results Summary

The proposed Dynamic Noise Adaptation (DNA) method addresses localization degradation primarily caused by time-varying motion uncertainty during normal driving. In practical mobile robot operation, such degradation commonly arises from acceleration and deceleration, wheel slip, surface irregularities, and frequent turning maneuvers, all of which amplify odometry errors even in static environments. Unlike reset-based approaches such as EMCL2, which utilize the non-penetration rate (NPR) only as a trigger for Expansion Resetting when localization reliability drops below a threshold, the proposed method continuously feeds NPR into the motion model. By dynamically scaling the motion noise parameters at every motion update, DNA increases particle dispersion before severe divergence occurs, thereby mitigating the accumulation of odometry-induced errors. This continuous adaptation mechanism enables smoother recovery from gradual localization degradation without relying on discrete reset events. In addition, when compared with virtual odometry generation based on NDT scan matching, as employed in improved AMCL, the DNA algorithm showed better performance under experimental conditions involving continuous robot motion rather than stationary conditions.

#### 4.5.5. Experimental Results Under Different Parameter Settings

The effects of different β and γ values are summarized in Table 4. The average RMSE values under different β settings were 0.069 m for β=0.5, 0.061 m for β=1.0, and 0.075 m for β=1.5. For γ, the average RMSE values were 0.071 m for γ=0.5 and 0.061 m for γ=1.0. Although the optimal β and γ values may vary depending on the environment, in environments such as the simulator setting, where sensor data are relatively accurate, increasing β excessively tended to increase the localization error, while using a value that was too small reduced the contribution of the proposed DNA mechanism. In addition, when γ was set to a smaller value, particle resizing was not sufficiently activated, resulting in increased localization error.

## 5. Conclusions

Accurate position estimation is essential for the autonomous navigation of mobile robots. Several registration-based localization techniques, such as ICP and NDT, have been investigated for this purpose. However, these approaches suffer from fundamental limitations, including high dependence on initial position estimates, increased computational load, and a lack of real-time capabilities in large-scale environments. This study addresses these limitations by proposing a dynamic noise adaptation technique applicable to MCL based on a particle filter. The proposed technique enhances accuracy and real-time performance by dynamically adjusting the magnitude of the noise parameter used in the MCL motion model, utilizing the NPR as a reliability metric. In addition, considering the possibility that particles may not exist at their actual positions when the particle distribution expands owing to increased noise, a technique for adjusting the particle count based on the NPR was integrated. The experimental results in the simulation environment demonstrate that the dynamic noise adaptation algorithm reduces the localization error with respect to the ground truth. In particular, the average RMSE was reduced by 0.0094 m. Although this numerical difference may appear small in absolute terms, it represents a meaningful improvement in applications that require centimeter- to millimeter-level accuracy, as discussed in the Introduction. Furthermore, similar performance improvements were observed in the real-world experiments.

## Figures and Tables

**Table 1:** Position MAE and RMSE for each trajectory and run in the simulator environment.

		MAE (m)			RMSE (m)		
Trajectory	Run	EMCL2	Virtual Motion	DNA	EMCL2	Virtual Motion	DNA
I-shape	1	0.062	0.124	0.061	0.073	0.129	0.061
I-shape	2	0.056	0.114	0.058	0.077	0.122	0.065
I-shape	3	0.052	0.109	0.052	0.074	0.113	0.065
I-shape	4	0.068	0.121	0.067	0.076	0.123	0.064
I-shape	5	0.064	0.125	0.047	0.073	0.129	0.061
L-shape	1	0.068	0.085	0.060	0.071	0.094	0.063
L-shape	2	0.067	0.084	0.060	0.070	0.091	0.062
L-shape	3	0.069	0.085	0.059	0.073	0.094	0.062
L-shape	4	0.069	0.085	0.059	0.072	0.095	0.061
L-shape	5	0.068	0.084	0.059	0.071	0.095	0.061
Square	1	0.084	0.101	0.083	0.064	0.109	0.057
Square	2	0.064	0.083	0.057	0.065	0.104	0.059
Square	3	0.064	0.083	0.057	0.066	0.105	0.060
Square	4	0.101	0.122	0.080	0.066	0.106	0.057
Square	5	0.098	0.121	0.087	0.066	0.109	0.058

**Table 2:** Position MAE and RMSE for each trajectory in the real-world environment.

		MAE (m)			RMSE (m)		
Trajectory	Run	EMCL2	Virtual Motion	DNA	EMCL2	Virtual Motion	DNA
I_shape	1	0.056	0.076	0.057	0.089	0.120	0.091
L_shape	1	0.058	0.084	0.039	0.082	0.109	0.048
U_shape	1	0.106	0.121	0.105	0.131	0.144	0.131
Unstructured	1	0.119	0.134	0.123	0.158	0.170	0.194
Unstructured	2	0.096	0.118	0.107	0.139	0.155	0.155
Unstructured	3	0.202	0.197	0.163	0.290	0.285	0.212
Unstructured	4	0.197	0.213	0.197	0.263	0.280	0.268
Unstructured	5	0.196	0.217	0.190	0.261	0.282	0.247

**Table 3:** Position MAE and RMSE for each run on the same trajectory.

		MAE (m)			RMSE (m)		
Trajectory	Run	EMCL2	Virtual Motion	DNA	EMCL2	Virtual Motion	DNA
I-shape	1	0.032	0.025	0.016	0.040	0.028	0.023
I-shape	2	0.100	0.088	0.077	0.115	0.108	0.102
I-shape	3	0.048	0.039	0.035	0.060	0.052	0.047
I-shape	4	0.083	0.073	0.065	0.108	0.102	0.098
I-shape	5	0.060	0.050	0.046	0.080	0.075	0.070
I-shape	6	0.064	0.053	0.050	0.090	0.085	0.080
I-shape	7	0.055	0.044	0.040	0.070	0.065	0.060
I-shape	8	0.041	0.032	0.028	0.052	0.040	0.035
I-shape	9	0.108	0.105	0.087	0.121	0.113	0.105
I-shape	10	0.071	0.060	0.056	0.100	0.095	0.090

**Table 4:** RMSE comparison under different β and γ values in the simulator environment.

		RMSE (m) Under β (γ=1)			RMSE (m) Under γ (β=1)	
Trajectory	Run	β=0.5	β=1.0	β=1.5	γ=0.5	γ=1.0
I-shape	1	0.073	0.061	0.076	0.072	0.061
I-shape	2	0.074	0.065	0.082	0.076	0.065
I-shape	3	0.077	0.065	0.079	0.074	0.065
I-shape	4	0.072	0.064	0.081	0.075	0.064
I-shape	5	0.069	0.061	0.074	0.070	0.061
L-shape	1	0.071	0.063	0.078	0.074	0.063
L-shape	2	0.072	0.062	0.076	0.073	0.062
L-shape	3	0.070	0.062	0.077	0.071	0.062
L-shape	4	0.069	0.061	0.075	0.072	0.061
L-shape	5	0.068	0.061	0.074	0.070	0.061
Square	1	0.066	0.057	0.071	0.067	0.057
Square	2	0.067	0.059	0.073	0.070	0.059
Square	3	0.069	0.060	0.074	0.071	0.060
Square	4	0.064	0.057	0.070	0.066	0.057
Square	5	0.068	0.058	0.072	0.067	0.058

## Data Availability

The data presented in this study are available on request from the corresponding author. The data are not publicly available due to privacy.
